# Accelerated Molecular Aging in Neighborhood Poverty: A Racial/Ethnic Comparison

**DOI:** 10.1093/geroni/igaf122.2876

**Published:** 2025-12-31

**Authors:** Jennifer Robinette, Jennifer Smith

**Affiliations:** Chapman University, Orange, California, United States; University of Michigan, Ann Arbor, Michigan, United States

## Abstract

In the US, racial/ethnic health disparities are undeniable and partially stem from residing in low SES neighborhoods. Associations between neighborhood SES and health may have some underlying molecular mechanisms reflected in the epigenome. Yet, neighborhood characteristics are not always experienced the same way for all residents, and questions remain regarding whether those most exposed to low SES neighborhoods build greater resilience to, or embody greater harmful and cumulative outcomes from, such neighborhoods. The present study tested the hypothesis that greater neighborhood poverty would relate to accelerated epigenetic aging on the Horvath, Hannum, PhenoAge, and GrimAge clocks, and that these associations would differ in strength by race/ethnicity. A national sample of 3,790 older non-Hispanic White, non-Hispanic Black, and Hispanic participants from the 2016 wave of the Health and Retirement Study and census tract poverty rates from the 2012-2016 American Community Survey were used to test these questions. Results of weighted linear regressions suggested that living in higher poverty neighborhoods was associated with accelerated PhenoAge and GrimAge (not Hannum or Horvath), adjusting for age, sex, and educational. After further considering smoking status, however, only the association with GrimAge remained. Investigating interactions with race/ethnicity suggested that poverty was more strongly associated with accelerated GrimAge among non-Hispanic White participants, but this racial/ethnic difference was completely reduced after considering smoking. These results further support the need for economic investment in low SES neighborhoods and encourage more research to determine the social and physical resources needed in such neighborhoods to attenuate accelerated epigenetic aging.

